# Comparative Analysis of Quality Attributes in Restructured Steam-Cooked Chicken, Pork, and Beef System as Affected by Freeze-Drying Duration

**DOI:** 10.3390/foods15060989

**Published:** 2026-03-11

**Authors:** Hongbo Yu, Long Chen, Zhengyu Jin

**Affiliations:** School of Food Science and Technology, Jiangnan University, 1800 Lihu Road, Wuxi 214122, China; yuhongbo@pec.com.cn (H.Y.); longchen@jiangnan.edu.cn (L.C.)

**Keywords:** rehydration capacity, LF-NMR, color, texture profile

## Abstract

This study systematically investigated the effects of freeze-drying on chicken, pork, and beef by examining pH, moisture content, rehydration capacity, water distribution, color, and texture profile at 2, 4, 6, 8, and 11 h. The pH values of all meats remained relatively stable within 5.6–6.2 throughout the drying process. Moisture content followed a “rapid dehydration-slower drying-stabilization” pattern, with pork retaining higher moisture during the mid-drying phase, while chicken and beef lost water more rapidly. The rehydration capacity increased with prolonged drying, with chicken showing the highest rehydration efficiency. Color changes were species-dependent. Specifically, chicken initially brightened before slight darkening, beef lost lightness with a temporary increase in redness, and pork gradually yellowed. Texture profiles also varied, with chicken maintaining relative stability throughout the drying process, beef showing temporary mid-drying hardness, and pork experiencing rapid declines in springiness and cohesiveness alongside fluctuating hardness. These findings provide valuable insights for optimizing freeze-drying protocols to preserve quality, functional performance, and sensory characteristics across different meat types.

## 1. Introduction

Freeze-drying is a dehydration process that removes frozen water through sublimation under low pressure [1]. Owing to its ability to minimize thermal degradation and oxidative damage, this technique is widely used in the food industry to preserve products such as fruit, vegetable, meat and instant food with improved stability and rehydration capacity [2]. Compared with conventional drying methods (hot air drying, spray drying, and vacuum drying ect.), freeze-drying better maintains the natural color, texture, and flavor, making it particularly valuable for high-quality protein-based foods [3,4,5].

Multiple processing parameters, including temperature, time, freezing or drying rate, and vacuum pressure, collectively govern the extent of moisture removal and the overall quality of freeze-dried products. Among these factors, temperature, time, and freezing/drying rates are closely coupled. Specifically, while the freezing/drying rate is jointly regulated by time and temperature, the freezing rate directly determines the formation characteristics and spatial distribution of ice crystals, thereby governing the subsequent drying rate [6]. Han et al. [7] noted that meat batter frozen at −70 °C required a longer drying time than that frozen at −20 °C. Similarly, the drying temperature has been reported to markedly affect the drying kinetics and the physicochemical characteristics of the final products [8]. In a related study, freeze-drying of chicken meat analogs at 20 Pa and 40 °C resulted in a higher structural integrity index than drying at 50 °C [1]. It was reported that drying above the eutectic temperature not only improved product quality but also shortened processing time and reduced energy consumption [9]. Prolonged drying time has been associated with increased thickness and hardness but decreased moisture content and elasticity in freeze-dried tilapia skin [10]. Vacuum level influences the moisture content of freeze-dried materials, with higher vacuum degrees leading to greater moisture retention [11,12]. Studies on fruit products have shown that freezing rate influences final moisture content, and that an optimal drying rate can significantly reduce processing time without compromising product quality [13,14]. In addition to the aforementioned processing parameters, factors such as loading capacity, sample dimensions, and material type also exert significant influence on the quality of freeze-dried products [2].

In the meat industry, freeze-drying has been extensively employed to produce lightweight, shelf-stable products with prolonged storage life and superior rehydration capacity [6]. However, the success of this technique strongly depends on the balance between sufficient water removal and the preservation of structural integrity. Insufficient drying often results in excessive residual moisture and poor microbial stability, whereas over-drying can induce structural collapse, protein denaturation, and lipid oxidation, ultimately leading to undesirable texture and flavor deterioration [3,6,15,16]. Although numerous studies have investigated the effects of processing parameters on the physicochemical properties of freeze-dried meat, the influence of drying duration remains ambiguous, and no consensus has been established regarding the optimal drying conditions. Moreover, previous research has primarily focused on single meat species, overlooking the inherent compositional and structural heterogeneity among different types of meat. Specifically, meats such as chicken, pork, and beef exhibit substantial differences in muscle fiber arrangement, fat distribution, and protein composition, all of which may distinctly influence their response to freeze-drying [6]. Nevertheless, systematic comparative studies examining how these meats behave under identical processing conditions remain scarce.

Therefore, the present study aimed to investigate the effects of different freeze-drying durations (2, 4, 6, 8, 11 h) on the pH, moisture content, rehydration capacity, the distribution of water, color, and texture profile of chicken, pork, and beef. It was hypothesized that drying duration would significantly affect the quality characteristics of meat and that the optimal freeze-drying time would differ among meat types due to their inherent compositional and structural differences. The findings are expected to provide insights for improving the quality control and process optimization of freeze-dried meat products.

## 2. Materials and Methods

### 2.1. The Preparation of Freeze-Dried Chicken, Pork, and Beef

Fresh chicken breast, pork hind leg, and beef were purchased from a local RT-Mart supermarket and immediately stored at −40 °C until use. Prior to processing, the samples were thawed naturally at room temperature, ensuring that the surface temperature remained below 15 °C. Each type of meat was then minced using a meat grinder (Midea, Foshan, China), with the temperature strictly maintained below 15 °C throughout the mincing and mixing processes. Crushed ice was added when necessary to prevent temperature elevation. The minced meat was then molded into slabs (50 × 50 × 8 mm) and subsequently cooked in a steam chamber at 105 ± 3 °C for 180 ± 5 s. After steaming, the samples were immediately cooled and rapidly frozen at −40 °C for at least 4 h. The frozen meat sheets were then cut into standardized cubes (12 × 12 × 8 mm) using a slicer (Aiboch, Jinhua, China) in a low-temperature processing room. The cubes were pre-frozen at −40 °C for another 4 h and then subjected to vacuum freeze-drying (TFDS0.25, Yantai True cold-chain Co, Ltd., Yantai, China) under a vacuum pressure of 90 Pa. The programmed shelf temperature was sequentially set at 30 °C for 0.5 h, 80 °C for 5 h, and 50 °C for 5.5 h under a constant chamber pressure of 90 Pa. Samples were collected at drying durations of 2, 4, 6, 8, and 11 h, respectively. All dried samples were vacuum-sealed, stored at −40 °C, and subsequently used for further analyses and characterization.

### 2.2. The Determination of pH

The determination of pH was performed following the method described by Kim et al. [17], with minor modifications. The freeze-dried meat samples were finely minced using a meat grinder, after which ten times the sample weight of potassium chloride solution (7.5 g/L) was added. The mixture was then homogenized using a homogenizer (Ultra-Turrax T18, IKA, Guangzhou, China) at 10,000 rpm for 60 s, with the temperature maintained at 20 ± 2 °C throughout the process. Prior to measurement, the pH meter (Delta 320, Mettler-Toledo GmbH, Schwerzenbach, Switzerland) was calibrated using standard buffer solutions to ensure accuracy.

### 2.3. The Determination of Moisture Content

The moisture content was determined according to the method described by Nie et al. [10]. Briefly, pre-dried aluminum dishes were weighed and recorded as m_0_. Approximately 3 g of minced samples subjected to different drying treatments were placed into the aluminum dishes, and the total mass was recorded as m_1_. The samples were then dried to a constant weight in a thermostatic oven at 103 ± 2 °C, after which the total mass was recorded as m_2_. The moisture content of each sample was calculated using Equation (1).

(1) $$\mathrm{moisture}\;\mathrm{content}(\%)=\frac{m_{1}-m_{2}}{m_{1}-m_{0}}\times 100$$

### 2.4. The Determination of Rehydration Capacity

The rehydration capacity was determined according to the method described by Li et al. [18] with slight modifications. Briefly, the empty beaker was accurately weighed and recorded as M_0_. Subsequently, the freeze-dried meat samples (12 × 12 × 8 mm) with different drying durations were placed into the beaker and weighed to obtain the pre-rehydration weight (M_1_). The samples were then immersed in 100 mL of hot water at 90 °C for 3 min, removed, allowed to cool slightly at room temperature. Excess surface moisture was gently removed using tissue paper. The rehydrated samples were weighed again, and the mass was recorded as M_2_. The rehydration capacity was calculated on the basis of dry matter.

(2) $$\mathrm{rehydration}\;\mathrm{capacity}\;(g/g)=\frac{M_{2}-M_{0}}{(M_{1}-M_{0})\;\times \;(1\;-\;\mathrm{moisture}\;\mathrm{content})}\;\times \;100$$

### 2.5. Low-Field Nuclear Magnetic Resonance (LF-NMR) Measurement

The distribution of water in the freeze-dried meat samples was determined following the method described by Zhu et al. [19] with minor modifications. Freeze-dried meat blocks were cut into approximately 2 g and placed into NMR sample tubes. Transverse relaxation times (T_2_) were measured using a MesoMR LF-NMR analyzer (Shanghai Niumag Analytical Instrument, Shanghai, China). Measurements were performed at 25 °C using the Carr-Purcell-Meiboom-Gill (CPMG) pulse sequence. Instrument parameters were set as follows: spectral width (SW) of 100 kHz, 90° (P1) and 180° (P2) pulse durations of 10 μs and 20 μs, respectively, number of data points (TD) of 150,028, echo time (TE) of 0.25 ms, number of echoes of 3000, and 32 scans per sample. The resulting data were processed using MultiExp Inv Analysis Software 4.09 (Shanghai Niumag Analytical Instrument Co., Shanghai, China) to obtain multi-exponential decay curves and to analyze the distribution characteristics of water within the samples.

### 2.6. The Determination of Color

The surface color of the freeze-dried meat was analyzed using a CR-410 colorimeter (Minolta Co., Osaka, Japan). Measurements were conducted under CIE standard illuminant C to obtain the lightness (L*), redness (a*), and yellowness (b*) values. The instrument was calibrated with a white reference plate (L* = 97.83, a* = −0.43, b* = 1.98) prior to each measurement to ensure accuracy and consistency of the results.

### 2.7. Textural Properties

Texture profile analysis was conducted following the method described by Kim et al. [17], with slight modifications. The rehydration method is consistent with that in Section 2.4. A texture analyzer (Stable MicroSystem Ltd., Surrey, UK) was used to measure hardness, springiness, cohesiveness, gumminess, chewiness, and resilience. The instrument parameters were set as follows: compression ratio of 40%, pre-test speed of 5.00 mm/s, test speed of 0.50 mm/s, post-test speed of 5.00 mm/s, trigger force of 5 g, test interval of 2.0 s, and using a P/36R probe.

### 2.8. Statistical Analysis

All experiments were conducted in triplicate using independently prepared samples. Results are presented as means ± standard deviations. Graphs were generated using Origin 2025b (OriginLab Corporation, Northampton, MA, USA). For each freeze-drying duration, differences among the three meat types (chicken, pork, and beef) were evaluated using one-way analysis of variance (ANOVA) with meat type as the fixed factor. When significant effects were detected, Duncan’s multiple range test was applied for post hoc pairwise comparisons. This analytical strategy was adopted to compare species-related differences under the same freeze-drying duration. Consequently, statistical comparisons were performed independently at each drying time point rather than using a full factorial model. All statistical analyses were performed using SPSS v23 (SPSS Inc., Chicago, IL, USA). Differences were considered statistically significant at *p* < 0.05.

## 3. Results

### 3.1. Changes in pH Values of Chicken, Pork, and Beef During Freeze-Drying

**Figure 1:**
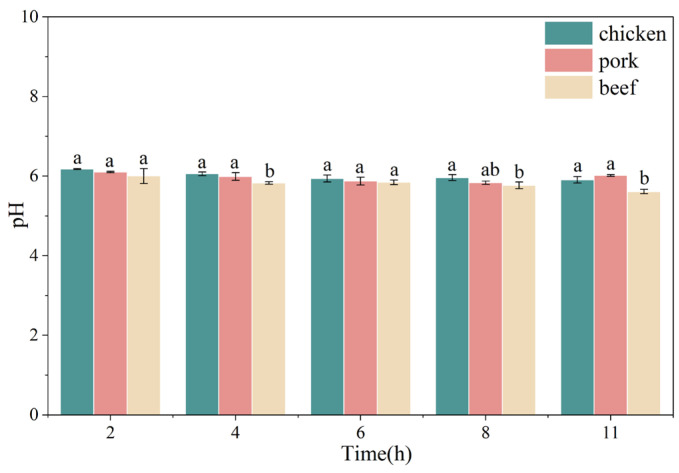
Changes in pH values of chicken, pork, and beef as affected by freeze-drying duration. Different letters indicated significant differences among meat types within the same freeze-drying duration (*p* < 0.05).

The pH value serves as a fundamental indicator of meat quality, directly influencing its functional properties, sensory characteristics, and storage stability [20]. As shown in Figure 1, the pH values of chicken, pork, and beef fall within the typical range for fresh meat (5.6–6.2), in agreement with the findings reported by Abdul et al. [21]. Chicken consistently exhibited the highest pH throughout processing, followed by pork and beef. Notably, the trajectory of pH change differed among the three meat types. Specifically, in the early stages of drying (2–4 h), a noticeable reduction in pH was observed across all samples, likely resulting from the concentration of free acidic groups as moisture content declined [19]. As drying progressed (6–8 h), the pH values of chicken and pork tended to stabilize, whereas that of beef continued to decline, reaching its minimum at 11 h. Interestingly, pork exhibited a slight pH rebound after 8 h of drying, possibly due to partial protein denaturation and the subsequent exposure of basic amino acid residues [22]. Overall, the distinct pH evolution patterns among chicken, pork, and beef may be attributed to differences in their biochemical composition, protein structure, and buffering capacity.

### 3.2. Changes in Moisture Content of Chicken, Pork and Beef During Freeze-Drying

**Figure 2:**
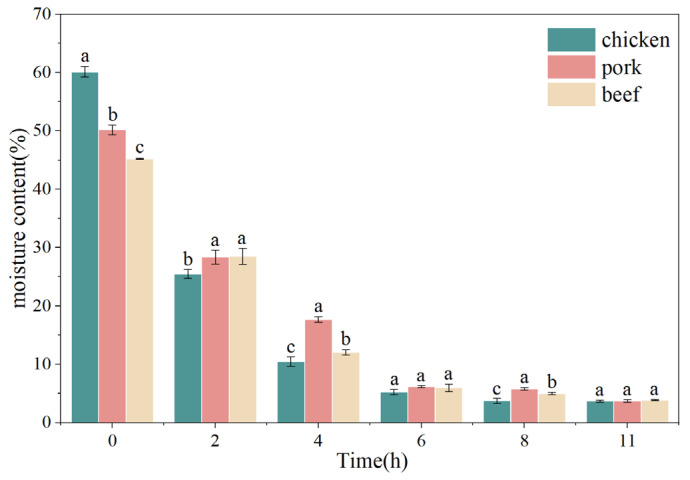
Changes in moisture content of chicken, pork, and beef as affected by freeze-drying duration. Different letters indicated significant differences among meat types within the same freeze-drying duration (*p* < 0.05).

As shown in Figure 2, the moisture content of chicken, pork, and beef all exhibited a clear declining trend during freeze-drying, although the rate of decrease and the final residual moisture varied among the different meats. During the initial stage (2–4 h), moisture content dropped rapidly, decreasing from approximately 25~30% to 10~18%, indicating rapid sublimation of ice crystals during primary drying. In the subsequent stage (4–6 h), the decline slowed markedly, reaching around 5~7% by 6 h. After 8–11 h, moisture levels stabilized, reflecting the slower removal of bound water. Differences in drying behavior were observed among the meat types. Throughout the process, pork consistently maintained slightly higher moisture levels than chicken and beef, particularly at 4 h, indicating a slower rate of water migration. Chicken and beef exhibited relatively rapid dehydration rates, approaching their final stable levels within 6 h.

All three meats followed a typical “rapid dehydration-slower drying-stabilization” pattern, indicating that under identical drying conditions, the samples primarily underwent two stages: rapid sublimation of free water followed by gradual removal of bound water [23]. The observed differences in moisture content among the meats likely reflect variations in tissue structure, fat content, and water-binding properties.

It should be emphasized that at 2 h of drying, the relatively high residual moisture content (approximately 25–30%) suggests that a substantial portion of frozen water likely remained within the matrix, indicating incomplete primary drying. At this stage, ice sublimation was still ongoing, and the sample cannot be considered to contain only adsorbed or protein-associated water. Therefore, the early drying period (2 h) primarily represents partial removal of ice rather than desorption of water tightly associated with protein chains. After 4 h of drying, when moisture content decreased markedly, primary sublimation was likely largely completed, and the remaining water was predominantly present as adsorbed or structurally associated water within the porous matrix. Under these conditions, further moisture reduction corresponds mainly to secondary drying, involving desorption of water bound to protein and confined within pore structures. In addition to protein–water interactions, compositional factors such as fat content may influence water removal behavior. Intramuscular fat can partially restrict ice crystal growth, alter pore morphology during sublimation, and obstruct capillary pathways, thereby affecting both moisture migration and subsequent rehydration characteristics. However, the relative contribution of lipid components requires further microstructural and compositional analysis for definitive clarification.

### 3.3. Changes in Rehydration Capacity of Chicken, Pork, and Beef During Freeze-Drying

**Figure 3:**
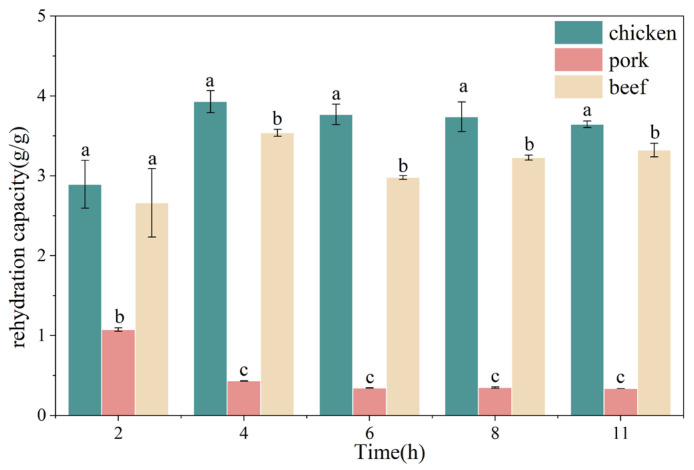
Changes in rehydration capacity of chicken, pork, and beef as affected by freeze-drying duration. Different letters indicated significant differences among meat types within the same freeze-drying duration (*p* < 0.05).

The rehydration capacity is widely used as a key indicator for evaluating the quality of dehydrated foods and the efficiency of drying methods [24]. As shown in Figure 3, chicken displayed the greatest rehydration capacity across the studied drying durations, increasing from 2.9 g/g at 2 h to a maximum of 3.9 g/g at 4 h, followed by a slight decline with extended drying (remaining 3.6–3.8 g/g from 6 to 11 h). Beef showed a similar but less pronounced pattern: rehydration capacity increased from 2.7 g/g at 2 h to 3.5 g/g at 4 h, decreased at 6 h (3.0 g/g), and then partially recovered at longer times (3.2–3.3 g/g at 8–11 h). In contrast, pork exhibited consistently low rehydration capacity and a monotonic decrease with time, dropping sharply from 1.1 g/g at 2 h to 0.4 g/g at 4 h and stabilizing at 0.3 g/g thereafter.

The initial increase in rehydration capacity observed in chicken and beef (2–4 h) may be primarily attributed to the development of a porous structure generated during ice sublimation [25,26]. During early primary drying, removal of ice crystals leaves behind interconnected capillary channels that enhance water penetration and absorption during subsequent rehydration. However, further extension of drying time did not continuously improve rehydration capacity. Instead, slight declines or stabilization at later stages (6–11 h) suggest that excessive dehydration may induce structural shrinkage, pore collapse, or increased protein aggregation, thereby limiting additional water uptake despite lower residual moisture content. The consistently poor rehydration performance of pork indicates a less favorable pore architecture or reduced structural stability during drying. In comminuted systems such as those used in this study, pore formation is governed predominantly by ice crystal templating and the ability of the protein matrix to maintain structural integrity during sublimation, rather than by intact muscle fiber organization. Differences in myofibrillar protein composition, connective tissue content, fat distribution, and susceptibility to denaturation may result in distinct microstructural stabilization behaviors among species. These variations likely contribute to differences in pore connectivity and capillary-driven water absorption, ultimately determining the observed species-dependent rehydration performance.

### 3.4. LF-NMR Analysis of Water Distribution During Freeze-Drying

**Figure 4:**
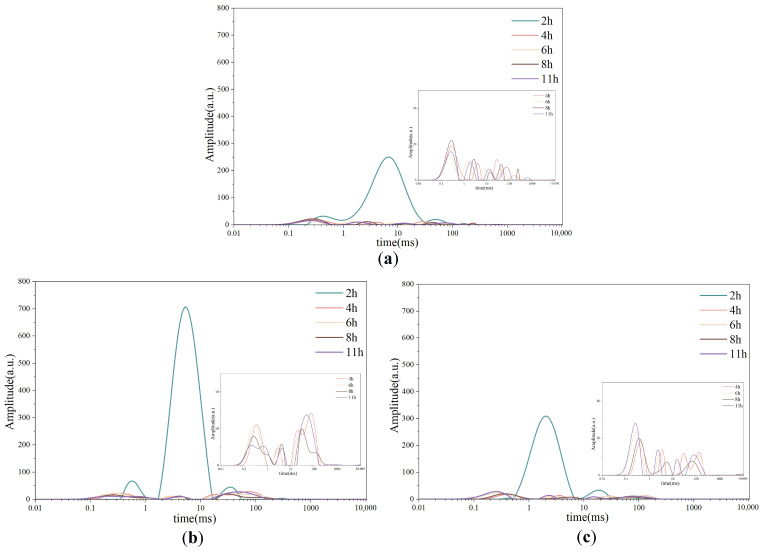
Changes in water distribution of chicken (**a**), pork (**b**), and beef (**c**) as affected by freeze-drying duration.

The LF-NMR transverse relaxation spectra (T_2_ distributions) of chicken (Figure 4a), pork (Figure 4b), and beef (Figure 4c) revealed distinct changes in water mobility during freeze-drying, reflecting the transformation of water from free to bound forms as drying progressed. According to the relaxation characteristics, the water in meat can be divided into four distinct states: strongly bound water (T_20_, 0.1–1 ms), weakly bound water (T_21_, 1–10 ms), immobilized water (T_22_, 10–100 ms), and free water (T_23_, 100–1000 ms) [19]. Among these, immobilized water (T_22_) is generally dominant in fresh muscle tissues [6]. The distribution and amplitude of these peaks provide valuable insights into the migration and transformation of water during the freeze-drying process.

At the initial drying stage (2 h), all samples exhibited a pronounced T_21_ peak, whereas the T_22_ and T_23_ peaks were comparatively weak, indicating that during freezing and early primary drying, free water had largely crystallized and begun sublimation, while a considerable fraction of immobilized water was still present within the frozen matrix at 2 h, consistent with the measured residual moisture content [27]. As drying progressed, the amplitude of the T_21_ component decreased sharply and was nearly absent by 4 h, reflecting progressive desorption of water associated with protein matrices during secondary drying rather than direct sublimation. Notably, species-specific differences were observed in the rate and extent of these transitions. Pork displayed the largest amplitude for bound water (T_20_ and T_21_) at 2 h, consistent with its higher initial moisture content. Moreover, pork retained a significant proportion of immobilized water (T_22_), which remained relatively stable over time, likely due to its unique composition and microstructure. The higher content of intramuscular fat and connective tissue in pork may contribute to this retention, as fat can physically obstruct water channels and connective tissue (primarily collagen) can bind water through its hydrophilic amino acid residues, forming a gel-like matrix that stabilizes immobilized water [28]. In contrast, beef, with a similar moisture content to pork at 2 h, exhibited T_2_ amplitudes resembling those of chicken, likely due to its denser structure and higher fiber content, which limit water mobility [29]. The abundant myofibrillar proteins in beef, while capable of binding water, may undergo more rapid denaturation or structural tightening during freeze-drying, leading to reduced water-holding capacity. Chicken, characterized by finer muscle fibers and lower connective tissue content, exhibited the smallest T_22_ amplitude, which nearly disappeared with increasing drying time. This behavior suggests that the more porous muscle structure of chicken facilitates water vapor diffusion, leading to a more rapid loss of immobilized water during sublimation [4].

### 3.5. Changes in Color of Chicken, Pork and Beef During Freeze-Drying

**Figure 5:**
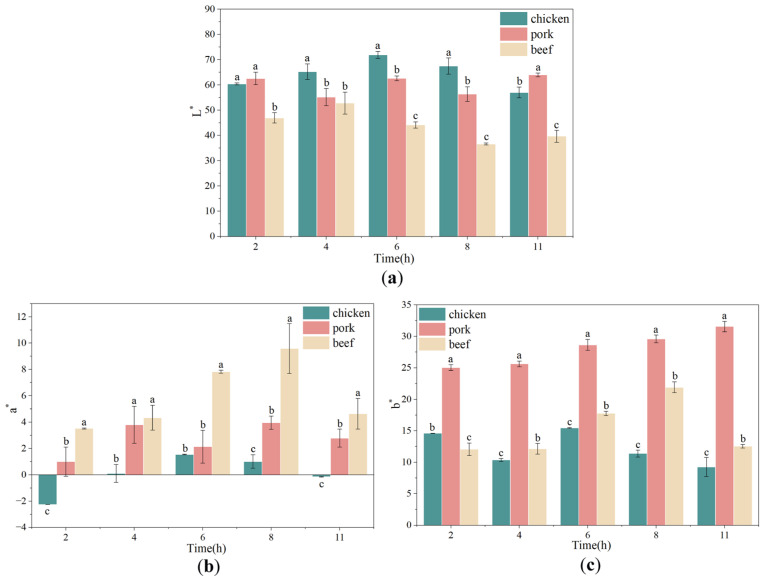
Changes in L* (Lightness, (**a**)), a* (Redness, (**b**)) and b* (Yellowness, (**c**)) of chicken, pork, and beef as affected by freeze-drying duration. Different letters indicated significant differences among meat types within the same freeze-drying duration (*p* < 0.05).

Instrumental color measurements revealed distinct and species-dependent patterns in lightness (L*), redness (a*) and yellowness (b*) as freeze-drying progressed. Lightness (Figure 5a) exhibited divergent behavior among species. Chicken L* increased from the initial time point to a maximum around mid-drying (4–6 h) before gradually decreasing toward the end. Pork showed moderate fluctuations, with a small trough early and a rebound in later stages. In contrast, beef displayed a continuous decline in L*, reaching the lowest values in the late drying period. The observed darkening is more plausibly associated with pigment concentration and oxidative transformations of myoglobin derivatives. At 2 h, no significant difference was observed between chicken and pork (*p* > 0.05, both being significantly brighter than beef. However, from 6 h to 8 h, the three species were significantly distinct (*p* < 0.05), following the order: chicken > pork > beef.

Redness (Figure 5b) varied markedly between species and over drying time. Beef presented the highest a* values overall, with a pronounced peak during mid-drying (6–8 h) followed by a decline. Pork exhibited modest increases with slight fluctuations, while chicken maintained low or slightly negative a* values, indicating minimal red hue. The mid-stage elevation of beef a* likely reflects concentration effects due to water removal and transient conversion of myoglobin to oxymyoglobin or other redox states, whereas subsequent decreases may result from progressive oxidation of heme pigments to metmyoglobin and other brownish derivatives under drying-induced oxidative stress [30,31]. At any given time point, the redness followed a consistent significant hierarchy: beef > pork > chicken (*p* < 0.05).

Yellowness (Figure 5c) increased steadily in pork, indicating accumulation of yellow-brown chromophores. Beef showed a mid-to-late rise in b*, peaking around 8 h, whereas chicken displayed smaller and more variable changes, with a mid-stage increase followed by decline. Comparison at equivalent time points revealed that pork consistently possessed the highest b^∗^ values (*p* < 0.05), while the differences between chicken and beef were time-dependent.

It should be noted that the moisture content of the samples decreased progressively throughout the freeze-drying process, which inherently affects the optical properties of the meat surface. Additionally, physical changes (porosity, surface scattering) and chemical transformations (pigment concentration, heme oxidation, lipid oxidation, and limited browning reactions) may also contribute to the observed color changes [23]. These species-specific responses suggest that a single freeze-drying schedule may not optimally preserve appearance for all meats: beef is prone to darkening, chicken achieves higher initial lightness but may lose brightness if over-dried. Consequently, color changes correlate with quality attributes such as consumer acceptance and perceived freshness, highlighting the need to optimize drying time, shelf temperature, and vacuum conditions for each meat type.

### 3.6. Changes in Texture Profile of Chicken, Pork and Beef During Freeze-Drying

**Figure 6:**
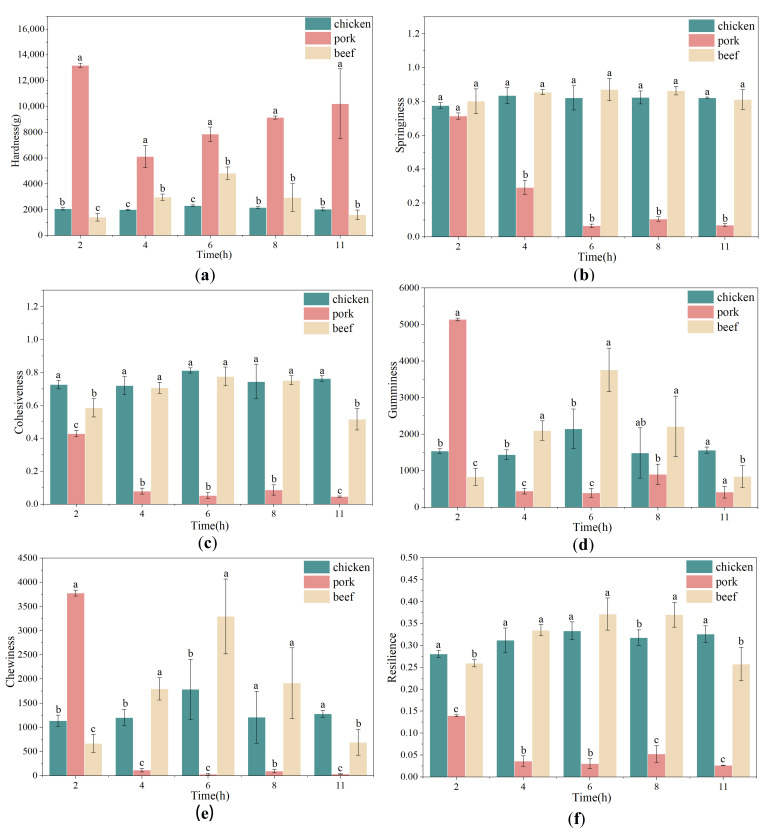
Changes in hardness (**a**), springiness (**b**), cohesiveness (**c**), gumminess (**d**), chewiness (**e**), and resilience (**f**) of chicken, pork, and beef as affected by freeze-drying duration. Different letters indicated significant differences among meat types within the same freeze-drying duration (*p* < 0.05).

The texture profile analysis (TPA) revealed pronounced, species-dependent changes in textural attributes across the freeze-drying process (2, 4, 6, 8 and 11 h). As shown in Figure 6, chicken exhibited the most stable texture parameters, pork underwent substantial and early structural changes, and beef displayed an intermediate pattern characterized by a transient mid-drying increase (6 h) followed by a decline.

Hardness (Figure 6a) remained relatively constant in chicken, suggesting that its structural integrity was minimally affected by the drying program. In contrast, pork exhibited extremely high hardness at 2 h (13,195 g), a sharp decrease at 4 h (6126 g), and a subsequent rise to high values by 11 h (10,222 g). Beef hardness peaked at 6 h (4828 g) before declining in later stages. These species-specific differences likely originate from distinct interactions among moisture removal, protein gel network behavior, connective tissue content, and fat distribution. For pork, the unusually high initial hardness may reflect a dense and compact matrix formed by partial water loss from a highly hydrated gel; subsequent pore development and partial collapse reduce hardness at intermediate times, whereas further desiccation promotes structural stiffening and increases hardness again [7]. The mid-stage hardness peak in beef suggests temporary reinforcement of the protein matrix due to concentration and partial aggregation of myofibrillar proteins, followed by later collapse or embrittlement.

Springiness (Figure 6b) and cohesiveness (Figure 6c) remained high and stable for chicken and beef, indicating preservation of elastic and resilient protein networks. In pork, however, both parameters decreased sharply after 2 h, implying severe early disruption of its elastic structure. Such deterioration may be associated with protein network disruption and microstructural collapse induced by progressive moisture removal, which transform an initially cohesive gel into a brittle, non-elastic matrix. The persistently low springiness and cohesiveness at later stages indicate irreversible loss of elasticity.

As composite indicators, gumminess (Figure 6d) and chewiness (Figure 6e) further magnified these species differences. Chicken showed moderate values with a small mid-stage peak, while beef exhibited a pronounced increase at 6 h, consistent with transient densification or aggregation of the protein matrix that elevates the mechanical energy required for deformation and mastication. Pork displayed extremely high gumminess and chewiness at 2 h, followed by an abrupt decline and sustained low values, paralleling its hardness and cohesiveness patterns and reflecting early structural breakdown that diminishes cohesive, chewable mass.

Resilience (Figure 6f) values increased slightly during mid-drying for chicken and beef and remained moderate, whereas pork showed a rapid decline to near-zero levels, with no recovery thereafter. The resilience trends align with springiness and cohesiveness and reinforce the conclusion that pork loses its elastic recovery capacity early in the freeze-drying process.

## 4. Conclusions

This study systematically investigated the effects of freeze-drying duration on the physicochemical and structural properties of chicken, pork, and beef. The pH values showed only minor fluctuations throughout the drying process, remaining within 5.6–6.2 for all three meat types. Moisture content followed a characteristic “rapid dehydration-slower drying-stabilization” pattern, with pork retaining relatively higher moisture during the mid-drying stage, while chicken and beef experienced more rapid water loss. Rehydration capacity increased progressively with extended drying time, with chicken exhibiting the highest water absorption efficiency, whereas pork and beef showed slower improvements. LF-NMR analysis indicated that most free and immobilized water was removed during the early drying stage; as drying progressed, bound water content gradually decreased in chicken and beef, whereas it remained largely stable in pork. In terms of color, chicken displayed early increases in brightness followed by slight darkening, beef experienced progressive loss of lightness with mid-stage enhancement of redness, and pork exhibited steady yellowing associated with oxidative changes. Texture analysis revealed that chicken maintained relatively stable structural properties, beef showed transient mid-drying strengthening before subsequent weakening, and pork underwent rapid loss of springiness and cohesiveness, accompanied by pronounced fluctuations in hardness. Overall, these results highlight that meat responses to freeze-drying are highly dependent on intrinsic compositional and microstructural characteristics, underscoring the need for species-specific drying protocols to optimize quality, rehydration performance, and sensory attributes. Future studies should validate these findings using intact muscle cuts to assess structural anisotropy effects, incorporate lipid oxidation kinetics, detailed microstructural characterization, and sensory evaluation, and further optimize chamber pressure and shelf temperature conditions for each species to establish species-specific freeze-drying protocols.

## Data Availability

The original contributions presented in this study are included in the article. Further inquiries can be directed to the corresponding author.
